# Analysis of Serotonin Molecules on Silver Nanocolloids—A Raman Computational and Experimental Study

**DOI:** 10.3390/s17071471

**Published:** 2017-06-22

**Authors:** Felicia S. Manciu, John D. Ciubuc, Emma M. Sundin, Chao Qiu, Kevin E. Bennet

**Affiliations:** 1 Department of Physics, University of Texas at El Paso, El Paso, TX 79968, USA; jdciubuc@miners.utep.edu (J.D.C.); emsundin@miners.utep.edu (E.M.S.); chaoqiu66@gmail.com (C.Q.); 2 Department of Biomedical Engineering, University of Texas at El Paso, El Paso, TX 79968, USA; 3 Border Biomedical Research Center, University of Texas at El Paso, El Paso, TX 79968, USA; 4 Division of Engineering, Department of Neurologic Surgery, Mayo Clinic, Rochester, MN 55905, USA; Bennet.Kevin@mayo.edu

**Keywords:** surface-enhanced Raman spectroscopy, theoretical calculations, serotonin detection, silver nanocolloids, biomaterials

## Abstract

Combined theoretical and experimental analysis of serotonin by quantum chemical density functional calculations and surface-enhanced Raman spectroscopy, respectively, is presented in this work to better understand phenomena related to this neurotransmitter’s detection and monitoring at very low concentrations specific to physiological levels. In addition to the successful ultrasensitive analyte detection on silver nanoparticles for concentrations as low as 10^−11^ molar, the relatively good agreement between the simulated and experimentally determined results indicates the presence of all serotonin molecular forms, such as neutral, ionic, and those oxidized through redox reactions. Obvious structural molecular deformations such as bending of lateral amino chains are observed for both ionic and oxidized forms. Not only does this combined approach reveal more probable adsorption of serotonin into the silver surface through hydroxyl/oxygen sites than through NH/nitrogen sites, but also that it does so predominantly in its neutral (reduced) form, somewhat less so in its ionic forms, and much less in its oxidized forms. If the development of opto-voltammetric biosensors and their effective implementation is envisioned for the future, this study provides some needed scientific background for comprehending changes in the vibrational signatures of this important neurotransmitter.

## 1. Introduction

5-hydroxytryptamine (5-HT), known as serotonin, is a neurotransmitter present in the central and peripheral nervous system. Although it was first found in the early 20th century as a smooth-muscle-stimulating substance in extracts prepared from gastrointestinal tract (GI) tissue [1], and later on as a vasoconstrictive substance in blood serum [2], it has been studied less than dopamine, another key neurotransmitter. Being found throughout the human body, 5-HT is implicated as having many physiological functions, which include its action as an immunomodulator for appetite, body temperature, sleep, anxiety, and circadian rhythm, just to mention its most important roles [1,2,3,4].

The intense research in biological psychiatry in the past few decades has demonstrated 5-HT’s influence on depression and suicide [5], as well as on the pathogenesis of Alzheimer’s disease. For example, studies on patients affected by Alzheimer’s show lower serotonin levels than those in control groups. Also, large numbers of neurofibrillary tangles were observed in the brains of the affected patients during autopsies and neurosurgery [6]. These accumulations, in the serotonergic cells in the raphe nuclei, were believed to be responsible for the induced neuronal death. The incidence of Alzheimer’s disease, with its most common form of senile dementia, has recently shown a dramatic rate of increase [7]. It has been estimated that, worldwide, 63 million people will be affected by 2030, and 114 million by 2050 [7]. Taking into account the selective neurodegeneration pattern that occurs in Alzheimer’s disease, it has been speculated that it is initiated by chronic attacks on axon terminals of long serotonergetic and noradrenergic neurons which connect the brain stem to blood capillaries [8].

Other studies of serotonin’s role in the pathophysiology of acute coronary artery disease syndromes demonstrate its increased concentration in patients with complex coronary artery lesions [9,10]. Besides clinical studies, animal model experiments of coronary artery stenosis and endothelial injury that attempted to reproduce clinical situations of unstable angina validated 5-HT’s role in initiating or maintaining cyclic reductions in coronary blood flow. Recurrent platelet aggregation and vasoconstriction [11,12], as well as alteration of coronary tone and development of myocardial ischemia [13], were reported.

Thus, research focusing on manipulation of serotonergic systems with drugs or by the deep brain stimulation (DBS) neurosurgical technique (which, being an invasive technique, is employed only in extreme cases) becomes of significant importance in promoting health and advancing treatments [12,14,15,16,17,18,19]. In this context, it is worth mentioning that unwanted irreversible oxidation of serotonin followed by the creation of neurotoxic byproducts, or its interaction with other neurotransmitters, have also been of concern in using appropriate drug administration and in its accurate monitoring by DBS [17,18,19,20,21,22,23]. For instance, tricyclic and monoamine oxidase inhibitor antidepressants were found to promote serotonin or noradrenaline function in the brain [22]. Although extensive application of selective serotonin reuptake inhibitors was efficient in increasing 5-HT’s neurotransmission, it also resulted in a suppression of dopamine neuron firing activity [23]. Furthermore, as the analyte underwent redox reactions during DBS measurements—each of which consists of a potential applied to an electrode and the measurement of current to generate a voltammogram (i.e., an N-shaped waveform showing a resting potential of +0.2 V, and for which voltage is scanned at a rate of 1000 V/s from +1.0 V to −0.1 V, then ultimately returning to and ending at +0.2 V)—oxidized forms of serotonin were indeed detected and reported [17,18,19]. Not only are these observations evidence of neural system complexity, but they also indicate the awareness that is needed in order to avoid unwanted health consequences.

The current study aims to elucidate all the detectable forms of serotonin through an optical approach, namely that of surface-enhanced Raman spectroscopy (SERS), which was first developed in the early 1970’s [24] and is well known to result in a dramatic enhancement in the optical signal of the investigated analyte [25,26,27]. Since this enhancement strongly depends on the roughness of the material used as a substrate, which can vary in scale from a few tens to hundreds of nanometers, as well as on the material type (usually a metal), both potentially being characteristics of electrodes currently employed in DBS, the analysis presented here could also shed some light on 5-HT monitoring, especially that of its unwanted oxidized forms. In addition, the experimental results presented in this work demonstrate the capability of SERS for detecting subphysiological levels of serotonin, as low as 10^−11^ molar. Through a combined and comparative approach between the outcomes of the density functional theory (DFT) computational method and this ultrasensitive, experimental detection technique, this study provides insights into this complex and not fully elucidated process. By making use of the advantages in being able to identify structural modifications of 5-HT through simulations and their consequent influence on Raman vibrational modes, discrimination between serotonic configurations is achieved and related valued predictions are discussed here. If the development of opto-voltammetric biosensors and their effective implementation is envisioned for the future, this study provides some needed scientific background for an accurate assessment and understanding of the results obtained.

## 2. Materials and Methods

Films of randomly packed silver nanoparticles (Ag NPs) were used in this case as substrates for SERS detection of serotonin. The Ag NPs were synthesized following previously reported procedures [28,29]. Reagents such as silver nitrate (AgNO_3_, >99%, from Sigma-Aldrich) and sodium borohydride (NaBH_4_, >99%, from Sigma-Aldrich), and citric acid trisodium salt dihydrate (C_6_H_5_Na_3_O_7_·2H_2_O, 99%, from ACROS) were used for such synthesis [28]. The chemical process, which is described in detail elsewhere [29], consists of first mixing 20 mL of 1% (w/v) citrate solution with 75 mL of ultrapure water, followed by addition of 1.7 mL of 1% (w/v) AgNO_3_ and 2 mL of 0.1% (w/v) NaBH_4_ solutions freshly prepared under heating at 80 °C and vigorous stirring. The reaction solution was then cooled to room temperature and purified many times to remove the excess of organic and unreacted impurities. Next, 90 μL of the liquid bearing these synthesized Ag NPs was mixed with 10 μL of 10^−10^ molar solution of 5-HT, which was obtained by successive dilution of the analyte in ultrapure water. The 100 μL mixture (which contains about 0.2 pg/100 μL of 5-HT) was sonicated for 20 s and drop-cast on clean cover slips, which upon drying formed dense, uniform thin films with a size distribution of Ag NPs ranging from 5 nm to 20 nm, as reported elsewhere [29]. At this very low concentration, the probability of achieving SERS enhancement by trapping the analyte in sufficient proximity of Ag NPs is very low; with the intent of increasing this probability, as well as analyte distribution uniformity, we directly mixed the Ag NPs with the serotonin solution before drop-casting.

An alpha300 R WITec system (WITec GmbH, Ulm, Germany) was used to perform the Raman measurements, which were acquired at ambient conditions in a backscattering geometry. The excitation of a 532-nm frequency-doubled neodymium-doped yttrium-aluminum-garnet (Nd:YAG) laser that was restricted to a power output of about 100 μW to avoid sample damage and a 20× objective (Olympus, Tokyo, Japan) were also used. The *WiTec Control 1.60* software with a Raman spectra time series acquisition capability was employed for achieving fast data acquisition, at 200 milliseconds per spectrum.

The quantum chemical density functional calculations were carried out using *Gaussian-09* analytical suite software. Energy optimization was first performed, followed by computation of Raman vibrational frequencies. Becke three hybrid exchange [30] and the Lee-Yang-Parr correlation functional, B3LYP [31], were employed in the current theoretical analysis. A Pople split valence diffused and polarized 6-311++G(*d,p*) basis set were used for calculations of all 5-HT molecular forms and an LanL2DZ basis set, which takes into consideration the pseudopotentials for metal atoms, was used for obtaining SERS simulation results of all serotonic forms in the proximity of silver dimers. Parsing of the *Gaussian-09* Raman output data with an in-house algorithm developed in C++ utilizing the Qt framework, and subsequent conversion through MATLAB version r2016a, was also performed. Next, conversion of Raman activities into relative Raman intensities was completed following a previously reported procedure [29,32,33,34] and taking into account the current 532 nm laser excitation. To enable data plotting, all Raman peak intensities were normalized by a factor of *f* = 1 × 10^−10^ and their shapes were modified by applying a Lorentzian band with a full width at half maximum (FWHM) of 7 cm^−1^.

## 3. Results and Discussion

The main advantage of Raman spectroscopy is that of providing detailed information about the molecular structure of the analyte under the study without the need for additional labeling. To this information, the use of confocal Raman microscopy adds the advantages of obtaining signals from very small volumes (i.e., femtoliter volumes ~1(μm)^3^) and of enabling spatially resolved measurements. Furthermore, the advancement of such technology currently facilitates very fast data acquisition, at millisecond scales, thus enabling measurements in time frames similar to those of physiological processes. The main challenge for the Raman technique is that of detecting trace amounts of substances. With the use of metallic nanostructures and SERS offering electromagnetic and chemical enhancements of the signal, this has been effectively surmounted [35,36]. Silver metallic nanoparticles have been used in the current experimental detection of 10^−11^ molar serotonin. Computational analysis by DFT was also compared against experimental data for an accurate and enhanced understanding of potential structural modifications of serotonin in the vicinity of Ag NPs.

**Figure 1:**
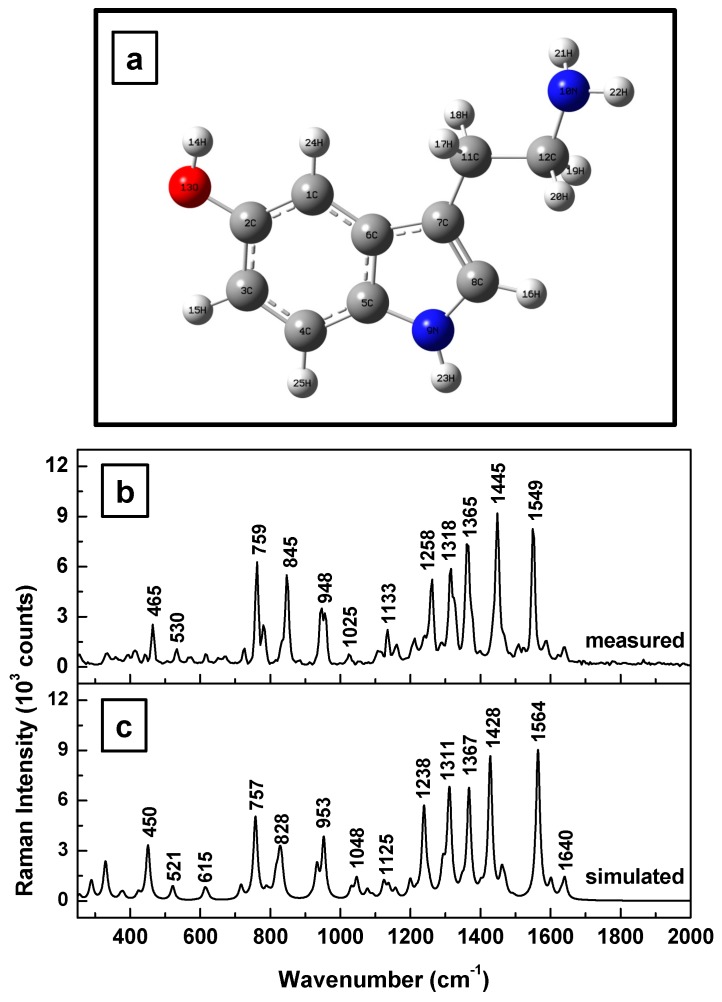
(**a**) Serotonin structural representation in neutral state after energy optimization. Red and blue colors were used for oxygen and nitrogen atoms, respectively. (**b**,**c**) Experimentally measured and theoretically calculated Raman vibrations of serotonin, respectively. The Raman spectrum was recorded for the standard 5-HT powder.

Calculations of 5-HT frequencies have been previously reported, especially those for solid serotonin [37]. However, for the purposes of cross-checking and confirmation of reproducibility, we first present in Figure 1a–c our simulation and experimental results for solid 5-HT (i.e., as purchased 5-HT powder). The optimized structural representation of this bioanalyte in its neutral form is shown in Figure 1a. The observed discrepancies of 9 ± 2 cm^−1^, on average, between the experimentally determined and the estimated Raman vibrational values in Figure 1b,c, respectively, demonstrate a relatively good agreement between these two outcomes, except for some variations in the peak intensities. The scaling factor of 0.98 applied to the simulated frequencies contributes slightly to better peak position agreements, specifically in reducing the discrepancies at higher frequencies. Scaling factors have been used in the literature to overcome systematic empirical errors originating from the force field constants employed in quantum mechanical approaches [32,38].

**Figure 2:**
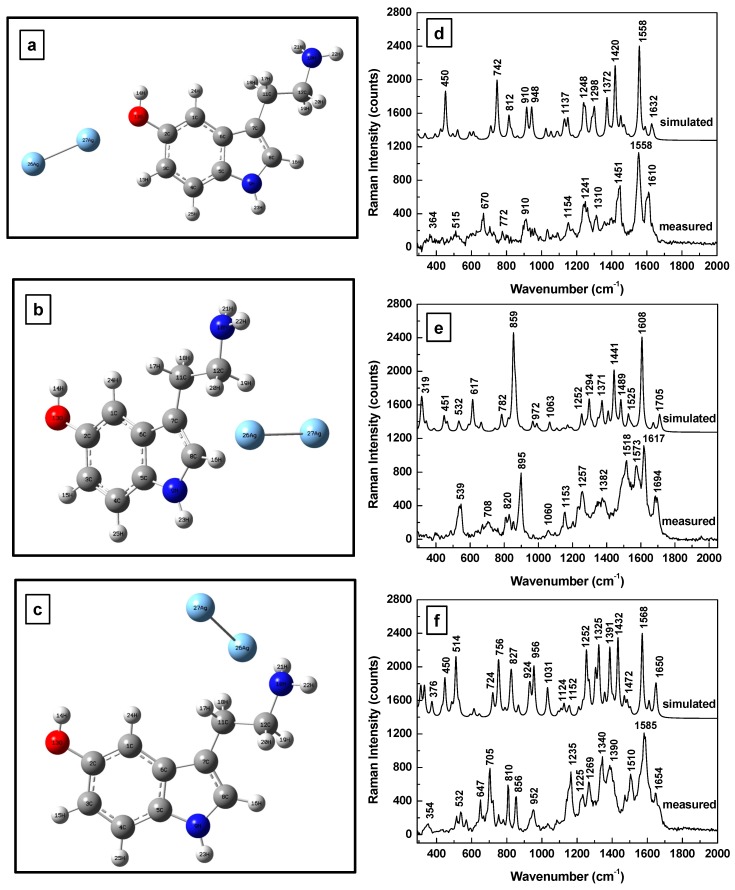
(**a**–**c**) Serotonin structural representation after energy optimization for silver dimer in the proximity of the hydroxyl group, between the NH and NH_2_ sites of the indole and amine groups, and between the OH and NH_2_ chemical bonds of the phenolichydroxyl and amine groups, respectively. (**d**–**f**) Theoretically estimated and experimentally recorded Raman vibrational spectra of neutral 5-HT associated with (**a**–**c**), respectively. The spectra are vertically translated for easier visualization and appropriately labeled.

The effect of the SERS environment on vibrational frequencies for neutral serotonin for both theoretical and experimental results is presented in Figure 2a–f. Again, for easier visual comparison, the energetically optimized structural representations of 5-HT in the close proximity of Ag NPs and with different positions of the silver dimer are shown in Figure 2a–c. A coplanar orientation of the silver dimer with respect to the molecular indole structure is observed in Figure 2a with proximity to the hydroxyl group; a perpendicular orientation is seen in Figure 2b with the dimer positioned between the NH and the NH_2_ sites of the indole and amine groups, respectively.

In the case of the silver dimer positioned between the OH and NH_2_ chemical functional groups (see Figure 2c), an optimum, minimum energy is obtained when it has a quasi-perpendicular orientation with respect to the indole plane, but is symmetrically tilted towards the phenolic hydroxyl group and the NH of the five member ring. As both of these sites are proton donors, they can accumulate more negative charge to electrostatically interact with the positive charge of the silver dimer. Thus, a potential explanation for all the observed silver dimer orientations could be associated with the internal polarizability of the 5-HT molecule (i.e., the internal dipole moment), with greater electronegativity associated with the hydroxyl functional group than with the amine group.

Corresponding comparisons of the theoretically predicted and experimentally obtained Raman results for each of the dimer positions shown in Figure 2a–c are presented in Figure 2d–f. The spectra are vertically translated for easier visualization and appropriately labeled. In addition to relatively good agreement between the simulated and measured Raman vibrations, as well as commonly observed Raman peaks around 1600 cm^−1^ for all of the above cases, these data also reveal the different signatures of the molecular interactions between the analyte and the surfaces of Ag NPs. For example, intense vibrational bands around 700 and 1400 cm^−1^ are observed for 5-HT physisorpted to the metallic surface through the OH of the phenol moiety, whereas a dominant vibration around 900 cm^−1^ is seen for serotonin interacting with silver through the NH and NH_2_ sites of the indole and amine functional groups in the geometrically perpendicular circumstance (see Figure 2b). For the silver dimer positioned between the hydroxyl and amine chemical groups, a multitude of strong Raman peaks are observed in Figure 2f, resembling a combination of these two previously discussed cases. The frequently observed vibration around 1600 cm^−1^ corresponds to the indole group’s deformation (i.e., C-H of the benzene ring and N-H stretching) and H-C-H scissoring; then, as is predictable, in all these cases hydrogen bonds are involved in the analyte interaction with the SERS metallic environment.

From the perspective of molecular polarizability, the dipolar components that are parallel to the silver surface are usually less enhanced [39]. When the overall induced molecular dipole moment results from the normal component of the electric field associated with the light excitation, which is along the dimer and perpendicular to the silver surface, dominantly in-plane vibrations are expected, such as those observed in Figure 2d. On the other hand, if the tangential field component induces the molecular dipole moment, out of plane vibrations such as those seen in Figure 2e and attributed to CH ring rocking are likely. Mixed Raman bands are expected for the case when a normal field is exciting a dipole with a strong component parallel to the metallic surface (see Figure 2f).

**Figure 3:**
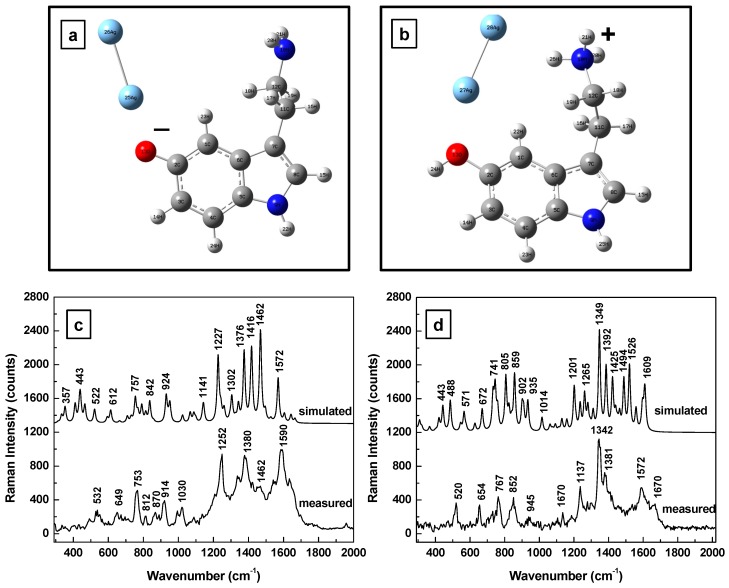
(**a**,**b**) Structural representations of the anionic and cationic forms of serotonin in the proximity of a silver dimer. (**c**,**d**) Theoretically calculated and experimentally measured Raman vibrations of the anionic and cationic forms of serotonin, respectively. The spectra are vertically translated for easier visualization and appropriately labeled.

Since the substance investigated here, 5-HT, is an ampholyte (its molecular structure contains a hydroxyl and an amine group) consideration of other serotonic forms such as anionic, 5-HT^−^, and cationic, 5-HT^+^, with their corresponding Raman spectra are presented in Figure 3a–d. Ionic dissociation of serotonin is expected when dissolved in water and it is likely to occur in the preparation of 10^−11^ M solutions. Although the current measurements were not performed in aqueous solution (for easier and more accurate comparison between the Raman spectra, as measurements in aqueous solutions introduce broadening of vibrational lines), during the drying process of the current sample preparation, some water molecules might not evaporate, remaining attached to the serotonin ions. Consequently, they could provide screening, hindering the re-association and neutralization of the charges. Since this screening is proportional to the high dielectric constant of water (i.e., ε = 80), it would be quite strong. Consequently, a decrease of Born energy to a lower value than that of the entropy related to dissociation of charges can occur. As such, when the value of the entropy is large, the 5-HT molecules remain charged and a dynamic equilibrium between the cationic and anionic forms of the serotonin molecules remains. Other contributing factors could be the creation of image charges in close proximity to the metallic Ag NPs or the induced thermal energy due to laser excitation. While thermal ionization is less likely, since a very low laser power of 100 μW was used during all measurements, we did not disregard it.

Obvious deformations of the serotonin molecular configuration through bending of the amine chain is observed for both the anionic and cationic forms shown in Figure 3a,b, respectively. Furthermore, a quasi-perpendicularity of the silver dimer to the 5-HT’s indole structure is seen in these situations, with the dimer orientation slightly opposing the end of the amine functional group for the anionic form (see Figure 3a) and electrostatically attracted towards the electronegative NH bond of the five member ring for the cationic form (see Figure 3b). There is a multitude of vibrations predicted for both ionic forms in Figure 3c,d, with dominant features ranging from 1200 to 1450 cm^−1^ for 5-HT^−^, and around 1350 cm^−1^ for 5-HT^+^. While Raman vibrational lines around 1200 and 1350 cm^−1^ were frequently measured, the occurrence of features around 1450 cm^−1^ was quite limited; vibrational lines around 1350 ± 20 cm^−1^ were more often encountered than those around 1450 ± 20 cm^−1^. This observation suggests a higher probability for the serotonin molecule to exist in anionic form than in the cationic one in the vicinity of the silver metallic surface. The Raman peaks around 1350 ± 20 cm^−1^ can be assigned to twisting deformation of H-C-H and the lines around 1450 ± 20 cm^−1^ to H-C-H wagging [37].

**Figure 4:**
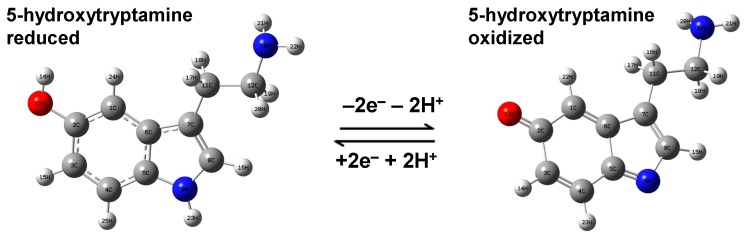
The redox process of serotonin consisting of the transfer of two electrons and two protons. The structural representations of oxidized and reduced 5-HT forms are presented and appropriately labeled.

**Figure 5:**
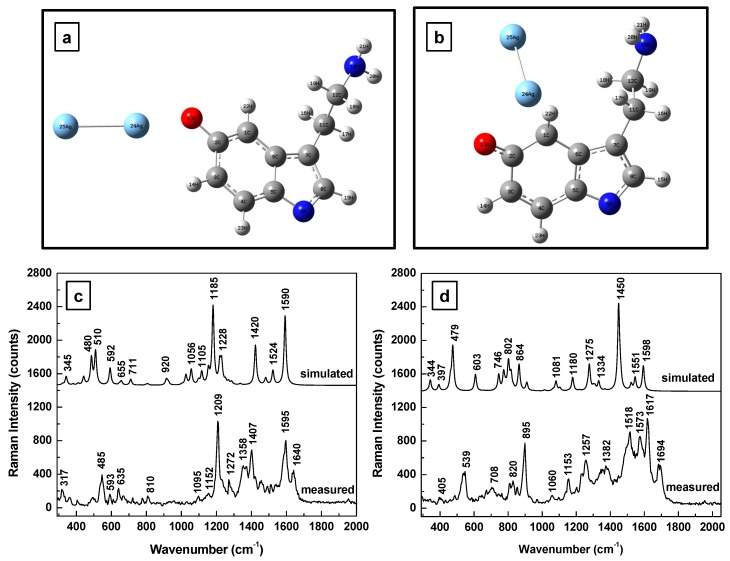
(**a**,**b**) Structural representations of oxidized forms of serotonin in the proximity of a silver dimer. (**c**,**d**) Theoretically calculated and experimentally measured Raman vibrations of the oxidized forms, (**a**,**b**), of serotonin, respectively. The spectra are vertically translated for easier visualization and appropriately labeled.

It is also worth considering and investigating the oxidized forms of 5-HT, which are known to result in a one-step redox reaction that is schematically presented in Figure 4, through the loss of two hydrogen atoms at the hydroxyl and NH chemical groups of the benzene and five member ring, respectively. These results are presented in Figure 5a–d. The reduced forms were already presented and discussed in Figure 1a–f. Again, bending of the amine chain of the oxidized form of 5-HT is observed for both configurations of silver dimer orientation, around oxygen or between the oxygen and the nitrogen atoms (see Figure 5a,b, respectively). The slightly tilted dimer position with respect to the indole plane and away from the two nitrogen atoms seen in the energetically stable configuration of Figure 5a results in the strongest theoretically predicted vibration around 1200 cm^−1^ in Figure 5c. A much more tilted dimer orientation is observed in Figure 5b, at almost a 45° angle to the indole plane. The predicted dominant vibration in Figure 5d at 1450 cm^−1^ that is scarcely found in measurements suggests that this configuration is less likely to occur; this potential adsorption process of serotonin on a silver surface through the lone pairs of oxygen and nitrogen was, however, first suggested by Song et al. [37].

## 4. Conclusions

With a perspective that stems from a desire to obtain a reliable understanding of vibrational assignments as compared with experimentally observed phenomena, the current study presents a detailed investigation of serotonin detection using Ag NPs as the SERS substrate. Not only is it more probable that serotonin will be adsorbed onto the silver surface at its hydroxyl/oxygen site than at its NH/nitrogen site, but the adsorption also occurs predominantly in the molecule’s neutral (reduced) form, followed probabilistically by its ionic forms, and is much less likely in its oxidized form. This observation is also supported by the commonly observed vibrations around 1200, 1350, and 1600 cm^−1^. The Raman feature around 900 cm^−1^, while still detectable at the current 10^−11^ molar concentration with the silver dimer positioned between the NH and NH_2_ sites of the indole and amine groups, respectively, and in a perpendicular configuration with respect to the indole plane, it is experimentally encountered more often at nanomolar or higher concentrations of serotonin [29]. As this configuration corresponds to a planar molecular orientation of 5-HT with respect to the silver surface (i.e., perpendicular to the silver dimer), this observation also suggests that, at higher concentrations, there is potential formation of multiple serotonic layers when the analyte is adsorbed at Ag NP surfaces. As a final note, if present, not only do the oxidized forms of 5-HT originate from other causes in the interaction of this analyte with other substances present in the body (such as a variety of metabolic acids), but the above considerations also explain why these forms are difficult to detect, both optically and electrochemically.
